# Editorial: Renal fibrosis and renal transplantation

**DOI:** 10.3389/fimmu.2026.1820123

**Published:** 2026-03-13

**Authors:** Cheng Yang

**Affiliations:** 1 Department of Kidney Transplantation, Zhongshan Hospital, Fudan University, Shanghai, China; 2 Shanghai Key Laboratory of Organ Transplantation, Shanghai, China; 3 Zhangjiang Institute of Fudan University, Shanghai, China; 4 Shanghai Institute of Medical Imaging, Shanghai, China

**Keywords:** biomarker, fibrosis, inflammation, kidney, transplantation

## 1 Introduction

Kidney transplantation remains the definitive treatment for patients with end-stage renal disease (ESRD), offering a significant survival advantage and improved quality of life compared to maintenance dialysis. However, the long-term success of transplantation is frequently hindered by chronic allograft dysfunction, which is characterized by the progressive accumulation of extracellular matrix—a process known as renal fibrosis. This “final common pathway” of injury is driven by a complex interplay of alloimmune responses, ischemia-reperfusion injury (IRI), metabolic disturbances, and sterile inflammation.

This Research Topic in Frontiers in Immunology, titled “Renal Fibrosis and Renal Transplantation,” brings together nine landmark papers—comprising original research, systematic reviews, and comprehensive literature syntheses—that shed light on the early prediction, molecular pathogenesis, and therapeutic targeting of graft injury.

## 2 Precision prediction: early biomarkers of graft outcome

The perioperative period is a critical window that often dictates long-term graft survival. Delayed graft function (DGF) is a common manifestation of severe IRI and serves as a precursor to chronic fibrosis. Identifying reliable, non-invasive biomarkers to assess donor organ quality is paramount.

Two studies in this Research Topic offer promising clinical tools for DGF prediction. Ma et al. identified donor urinary uridine diphosphate-glucose (UDP-Glc) as a novel damage-associated molecular pattern (DAMP) that independently predicts DGF. Their study demonstrates that elevated UDP-Glc levels correlate with more severe kidney damage and poorer graft recovery, providing surgeons with a stable, easy-to-detect biomarker for evaluating renal quality before implantation. Complementing this work, Leng et al. focused on cold-inducible RNA-binding protein (CIRBP) in donor plasma. Their findings reveal that donor CIRBP concentrations in the DGF group are significantly higher than in the immediate graft function group, establishing CIRBP as a potent indicator of the extent of pre-transplant injury and its subsequent impact on long-term plasma creatinine levels.

## 3 Molecular drivers: from acute injury to chronic fibrosis

Understanding the transition from acute kidney injury (AKI) to chronic kidney disease (CKD) is essential for preventing fibrosis. Peng et al. utilized Weighted Gene Co-expression Network Analysis (WGCNA) and machine learning algorithms to identify *ALDH2* as a pivotal target in kidney transplantation-related AKI. Their animal experiments demonstrated that increasing ALDH2 expression alleviates renal function impairment by inhibiting the MAPK signaling pathway, thereby reducing oxidative stress and inflammation—two primary triggers of fibrotic progression.

The role of the immune microenvironment in the chronically injured kidney is further explored by Atwood et al., who investigated the formation of Tertiary Lymphoid Tissues (TLTs). Their research highlights that the mTORC1 signaling pathway, specifically through p-S6 activity within the TLTs, drives the proliferation of immune cells and the growth of these ectopic lymphoid structures. This study provides a strong rationale for using mTOR inhibitors to disrupt the maintenance of chronic inflammatory niches that foster fibrosis.

Moreover, the mechanism of cell death itself contributes to the inflammatory milieu. Gao et al. provided a comprehensive review of Gasdermins (GSDMs) and their role in pyroptosis within the context of CKD. They detailed how GSDMD- and GSDME-mediated cell membrane rupture leads to the release of pro-inflammatory cytokines like IL-1β and IL-18, reinforcing the “vicious cycle” of inflammation and fibrosis in diseases such as diabetic nephropathy and lupus nephritis.

## 4 Therapeutic frontiers: targeting metabolic and inflammatory pathways

Traditional immunosuppressive protocols are often associated with metabolic toxicities that can paradoxically accelerate graft loss. Wang et al. performed a systematic review and meta-analysis comparing Belatacept-based regimens with calcineurin inhibitors (CNIs). Their analysis confirmed that Belatacept significantly reduces the risk of post-transplant diabetes mellitus (PTDM), offering a more favorable metabolic profile that could enhance long-term cardiovascular and graft outcomes.

Further expanding the therapeutic horizon, Peng et al. explored the potential of GLP-1 and glucagon receptor (GCGR) dual agonism. Their study found that the dual agonist TB001 ameliorates kidney allograft fibrosis in rat models by improving lipid metabolism. By upregulating CPT1A and inhibiting the TGF-β1/Smad and PKC pathways, TB001 directly addresses the metabolic-fibrotic axis, suggesting that targeting metabolic syndrome components can be a viable strategy for managing chronic allograft dysfunction.

Finally, the systemic and local inflammatory responses are addressed in two major reviews focusing on cytokine signaling. Gubernatorova et al. analyzed the renoprotective potential of IL-6 inhibitors. Given IL-6’s pleiotropic role in mediating T-cell and B-cell responses, neutralizing IL-6 signaling pathways represents a promising approach to treating both acute rejection and chronic fibrosis. Similarly, Bogdanova et al. reviewed the targeting of the IL-1 family. They emphasized how the balance between pro-inflammatory (IL-1α, IL-1β) and anti-inflammatory (IL-1Ra) components is disrupted during renal disease progression. Pharmacological inhibitors of IL-1 offer a potential niche for preventing the sterile inflammation that drives interstitial fibrosis.

## 5 Summary and perspective

The collective findings in this Research Topic underscore that renal fibrosis in transplantation is not a solitary event but the culmination of early donor injury, persistent immune activation, and metabolic maladaptation.

As Guest Editors, we hope that this Research Topic inspires further research into the integrated management of renal transplantation. By combining advanced diagnostics with multi-target therapies, we can move closer to the goal of “one transplant for life,” ultimately minimizing the burden of fibrosis and maximizing the gift of life for our patients.

